# Evidence for Skill Level Differences in the Thought Processes of Golfers During High and Low Pressure Situations

**DOI:** 10.3389/fpsyg.2015.01974

**Published:** 2016-01-07

**Authors:** Amy E. Whitehead, Jamie A. Taylor, Remco C. J. Polman

**Affiliations:** ^1^School of Sport Studies, Leisure and Nutrition, Liverpool John Moores UniversityLiverpool, UK; ^2^School of Psychology, University of Central LancashirePreston, UK; ^3^School of Psychology, Bournemouth UniversityBournemouth, UK; ^4^Institute of Sport, Exercise and Active Living, Victoria UniversityMelbourne, Australia

**Keywords:** Think Aloud, verbal protocol, motor task, golf, cognitive processes, expertise, reinvestment, stress

## Abstract

Two studies examined differences in the cognition of golfers with differing levels of expertise in high and low pressure situations. In study 1, six high skill and six low skill golfers performed six holes of golf, while verbalizing their thoughts using Think Aloud (TA) protocol. Higher skilled golfers’ cognitive processes centered more on planning in comparison to lower skilled golfers. Study 2 investigated whether thought processes of golfers changed in response to competitive pressure. Eight high skill and eight moderate skilled golfers, completed a practice round and a competition round whilst verbalizing thoughts using TA. To create pressure in the competition condition, participants were instructed that monetary prizes would be awarded to the top three performers and scores of all golfers would be published in a league table in the club house. When performing under competitive pressure, it was found that higher skilled golfers were more likely to verbalize technical rules compared to practice conditions, especially during putting performance. This shift in cognition toward more technical aspects of motor performance was strongly related to scores on the Decision Specific Reinvestment Scale, suggesting individuals with a higher propensity for reinvestment show the largest changes in cognition under pressure. From a practical perspective, TA can aid a player, coach or sport psychologist by allowing thought processes to be identified and investigate a performer’s thoughts when faced with the pressure of a competition.

## Introduction

Recent research (Whitehead et al., 2015) has encouraged the use of Ericsson and Simon’s (1980) Think Aloud (TA) protocol to record the cognitive processes of sports men and women during task performance. TA involves individuals continuously reporting their thoughts during the performance of a task (Ericsson and Simon, 1993). Little research has used TA to investigate cognition in sport. However, there are several studies employing TA in other domains such as chess (Gobet and Charness, 2006), medicine (Ericsson, 2004, 2007), nursing (Aitken and Mardegan, 2000), Scrabble (Tuffiash et al., 2007), and algebra tasks (Cook, 2006).

Whitehead et al. (2015) argued, that if the thoughts of sport performers were recorded during the performance of a task using TA, then this could be used to better understand cognition in sporting events. Much of the research investigating cognition in sport employs either laboratory based studies using simulated sport situations that lack ecological validity (e.g., McRobert et al., 2007) or retrospective methods for recording thoughts (e.g., Macquet, 2009; Cotterill et al., 2010; Mulligan et al., 2012). Laboratory based studies that employ unrealistic sports tasks or situations are limited in advancing a clear understanding of the cognitions of sportsmen and women if tasks are not representative of true sport tasks. Similarly, there are limitations with studies that employ retrospective methods, where participants are interviewed after the completion of a sports event to ascertain their thoughts during that event. This approach may lead to memory decay (Ericsson and Simon, 1980; Nicholls and Polman, 2008) or distorted reports by knowledge about task success (Brown and Harris, 1978). For example, Whitehead et al. (2015) showed that the use of TA in golf produces richer verbal data regarding decisions than retrospective methods. In this study, participants’ recall of thoughts after the completion of a round of golf had low levels of similarity (38–41%) with what was verbalized in event using TA.

There are concerns that asking performers to TA may disrupt motor task performance (Calmeiro and Tenenbaum, 2011), since TA may promote a more inward focus of attention that could lead to reinvestment in explicit rules (Whitehead et al., 2015). However, recent research has shown that golf putting performance was not negatively impacted by asking participants to TA when putting (Arsal, 2013; Whitehead et al., 2015). Whitehead and colleagues also found no clear evidence for reinvestment when thinking aloud, since golfers verbalized very few thoughts about technical aspects of the putting stroke when thinking aloud. The evidence to date, suggests TA may be a useful methodology for exploring cognition of sport performers and does not harm performance.

Think Aloud may be a particularly useful method for investigating differences in the cognition of sports performers of various levels of ability. Significant debate still exist around which theory best explains how motor skills are acquired (e.g., Rose and Christina, 2006). However, a number of models have been proposed which mainly describe the behavioral changes that occur during the learning process. The most relevant model for the present study is that put forward by Fitts and Posner (1967) which considers both cognitive and behavioral aspects and proposes that a learner moves through three stages of learning, from cognitive, associative to autonomous. Other models, such as Gentile’s (1972) two stage model also provides a similar process of skill development. However, this model emphasizes the environment and how this influences goal achievement (mainly behaviourally oriented). The ecological or dynamical systems perspective (e.g., Kam et al., 1990) focusses on the changing nature of perception and action dynamics of the environment with little emphasis on cognitions.

According to Fitts and Posner (1967) at the cognitive stage a novice’s performance is based on a set of cognitive rules and performance is controlled in a step-by-step fashion. The associative phase is where the performer starts to gain a better understanding of the task and movement patterns become more refined. Finally, the autonomous phase is where skill execution is fully automatic and conscious attentional control is no longer required to execute a particular action. What a performer cognitively attends to throughout performance will differ depending on where they are on this skill continuum. To capture these cognitions, TA could be used. For example, Calmeiro and Tenenbaum (2011) demonstrated how TA could be used to capture differences in the cognitions of three experienced and three novice golfers when performing a putting task. Findings revealed that, experienced golfers spent more time than beginners assessing conditions and planning prior to a putt and verbalized more diagnostic-related thoughts and planning of the next putt following the putt execution. This finding was consistent with McPherson (2000) and suggests that information concerning past performance outcomes is used more by experienced players to diagnose and update subsequent performance strategies. In contrast, low skilled golfers focused more on the technical aspect of the putt. Calmeiro and Tenenbaum (2011) suggest that in line with previous models of skill acquisition, experienced players did not engage in technical instruction, which might indicate a higher degree of automaticity of motor control where the performer’s skill is controlled by procedural knowledge and in the automatic phase. In comparison, the verbalization of more technical instructions by novices, indicates skill execution is controlled more by declarative knowledge that is attended to in a step-by-step fashion (Fitts and Posner, 1967; Anderson and Lebiere, 1998).

Calmeiro and Tenenbaum’s (2011) research was an original and novel investigation, however, the expertise level and the sample size of the two groups are questionable. Particularly, in the high skilled (HS) group there were three performers with handicaps of 0, 13, and 18. A handicap of zero and 18 represent an extremely different level of performance. In addition, whilst this study provides an important insight into skill level differences in decision making of golfers, the very small sample size limits the generalizability of findings. Additionally, the study was conducted in an artificial setting and therefore, there is a necessity for this type of work to be conducted with larger sample sizes and in a more ecologically valid environment, such as a real life golf course.

It is important to note that skilled performers in the autonomous phase of learning may still encounter skill breakdown and skill regression when performing in what they may perceived as a high stress situation (Masters, 1992). This has also been termed as choking, which refers to the decrease in athletic performance because of disruption in the execution of habitual processes under situations of stress or pressure (Beilock and Gray, 2012). Masters (1992) has also termed this type of behavior as reinvestment. Reinvestment theory (Masters, 1992) predicts that during times of stress, changes occur in cognitive processing. That is, the automaticity of a task becomes undone or disrupted as the performer tries to control a task or action consciously with declarative knowledge. According to Masters (1992), skilled performers may regress to an earlier stage of learning when performing a task in a stressful situation. A disruption in performance occurs when an ‘integrated’ real time control structure that can run as an uninterrupted unit (e.g., a professional golfer driving off the tee) is broken down back into smaller, separate independent units, similar to how it was originally attended to in a step-by-step fashion during the early stages of skill learning. This in turn slows down performance as each component is run separately instead of all together. As a result there is a gap in each unit which creates more room for error, which would not be present in the integrated autonomous structure (Masters, 1992; Beilock and Carr, 2001). Therefore, if a performer’s motor movement is broken down, then the cognitive process of a performer would be more likely to be focussed on the internal mechanics of a skill rather than external factors such as planning or where to aim. For example, Nicholls and Polman (2008) found that when under stress, high level golfers reverted to a high frequency of swing thoughts, which are technical thoughts about their performance and this was evident when they were asked to TA and verbalize their thought processes. The use of TA may help to further the understanding of choking and reinvestment in sport (Kinrade et al., 2015).

Masters et al. (1993) suggested that reinvestment could be a characteristic of personality and a 20-item scale was initially developed to measure reinvestment. This scale was found to correlate with performance decrements on motor tasks under conditions of high stress (e.g., Chell et al., 2003; Jackson et al., 2006). However, the scale was criticized since it did not focus specifically on movement reinvestment and therefore lacked face validity (Jackson et al., 2006). Masters et al. (2005) subsequently developed a two factor movement specific reinvestment scale. The first factor of the scale measured movement self-consciousness, which reflected concern about style of movement and making a good impression in public. The second factor measured conscious motor processing, which reflected the contemplation of the process of movement (Masters and Maxwell, 2008). This scale has been used primarily in clinical studies rather than sport contexts. For example, medical students scoring high on movement specific reinvestment tended to show slower and less efficient performance on a laparoscopy task when under time pressure than students scoring low in reinvestment (Malhotra et al., 2012). Further research is necessary to understand how movement specific reinvestment relates to changes in cognition when performing a sports task under pressure.

More recently Kinrade et al. (2010) introduced the concept of decision making reinvestment. They developed a Decision-Specific Reinvestment Scale (DSRS) comprising of six items specific to the conscious monitoring of the process involved in making a decision (decision reinvestment). A second factor which makes up the scale is decision rumination, which focusses on negative evaluations of poor decisions. When validating this scale, Kinrade et al. (2010) found that the scores of 59 skilled team sports players correlated highly with coaches ratings of players’ tendency to choke under pressure. Further work by Kinrade et al. (2015) indicated global scores on the DSRS predicted actual choking under pressure on a computer-based complex basketball decision making task. Specifically, decision rumination predicted the breakdown of decision making under pressure on a complex version of the task, but predicted faster completion times on a simple versions of the task when under pressure. This finding indicates decision making reinvestment only predicts performance breakdown under pressure on complex tasks. However, the computer based nature of the task lacked ecological validity. Therefore, further work is necessary to investigate changes in decision making under pressure on real sport tasks.

The present paper uses TA to further develop the understanding of the cognition of sport performers in event. Study 1 aims to extend the work of Calmeiro and Tenenbaum (2011) by investigating the differences in decision making processes between six high and six lower level golfers over six full holes of golf, using the TA methodology. This extends the previous work, as the study considers the whole game of golf, rather than just one (putting). Based on Fitss and Posner’s model of learning it was predicted that skilled golfers would verbalized more thoughts around planning prior to shot execution and evaluation post shot execution whereas, lower skilled golfers would verbalized more technical thoughts. In Study 2, the aim was to investigate whether stress through the introduction of a competition with monetary prizes, influences the thought process of golfers of differing levels of skill; eight high and eight intermediate. Specifically, the study aims to examine if there is a tendency for reinvestment among higher skilled golfers, when playing in a high stress situation, as evidenced by a greater focus on technical aspects of their motor performance. It was predicted that higher skilled golfers would verbalize more technical thoughts during competition in comparison to practice Furthermore, Study 2 investigated whether measures of propensity for movement-specific reinvestment and decision-specific reinvestment are related to greater focus on technique when under stress. It was predicted that those who scores higher on movement reinvestment and decision specific reinvestment would revert to verbalizing more technical thoughts when in a pressured situation such as a competition compared to those scoring low.

## Study 1

Previous literature, (Thomas and Over, 1994; Calmeiro and Tenenbaum, 2011) has provided evidence that higher skilled golfers spend more time planning and evaluating their shots. Whereas lower skilled golfers spend more time devoted to the technical mechanics of their performance. Such findings are in line with Fitts and Posner’s (1967) model of skill development, with lower skilled performers engaging in more conscious cognitive control of motor task performance. However, much of this research is based on retrospective reports (Thomas and Over, 1994) or conducted in an artificial setting, with a focus only on the putting aspect of the game of golf (Calmeiro and Tenenbaum, 2011). Therefore, Study 1 aims to further previous research by exploring the decision making process using TA when playing on a real golf course. Based on previous research (Thomas and Over, 1994; Calmeiro and Tenenbaum, 2011) and Fitts and Posner’s (1967) model, it was predicted that skilled golfers would focus more on pre-shot planning, whereas less skilled golfers would be more focused on the technical elements of playing a shot. Furthermore, it was predicted that skilled golfers would verbalize more thoughts in the evaluation of a shot, since skilled and experienced performers have been found to describe and evaluate performance more than beginners in previous work (McPherson, 2000; Calmeiro and Tenenbaum, 2011).

### Method

#### Participants

Participants were six male HS golfers (age: *M* = 16.33, *SD* = 0.51, handicap: *M* = 4.16, *SD* = 0.75) and six male low skilled golfers (age: *M* = 26.33, *SD* = 8.52, handicap: *M* = 20.16, *SD* = 5.34). The study and protocol was approved by the University of Central Lancashire ethics committee written consent was provided prior to participation in the study.

#### Materials

Each golfer played with their own golf clubs on the same six holes (1 par 5, 3 par 4’s and 2 par 3’s) of the same golf course in North East England. Participants’ verbalizations were recorded using a Sennheiser USA ENG G3 wireless digital voice recorder. The recording device was placed in the pocket of the participant, with a wire running inside the shirt connecting to a microphone attached to the collar.

#### Procedure

Participants were initially briefed on how to conduct TA (Ericsson and Simon, 1993). Participants then took part in a series of TA exercises which included (1) counting the number of dots on a page (2) an arithmetic exercise and (3) an anagram problem-solving task, and were asked to TA when completing the exercises. Each of the golfers then played six holes of golf accompanied by an experimenter. Participants were instructed to verbalize their thoughts continuously throughout the six holes apart from when they were executing their shot. It is important to note that participants were not instructed to verbalize during shot execution to reduce any interference with motor movement (Schmidt and Wrisberg, 2000). If they were silent for a period of longer than 20 s they were asked to resume thinking aloud.

#### Data Analysis

Each participant’s verbal reports from TA were transcribed verbatim. Following checks for relevance and consistency each transcript was subjected to a line by line content analysis (Maykut and Morehouse, 1994) by the first author to identify verbalizations which related to the decision making process of each shot played. Each verbalization was then grouped according to a modified version of the coding scheme (see **Table 1**) developed by Calmeiro and Tenenbaum (2011). The second author coded a random sample of verbal data; the inter-rater agreement was 95%.

**Table 1:** Themes used to code verbalizations.

Theme	Description	Example of raw data quote
Gathering information	Searching for relevant characteristics of the environment	“There’s a break left,” “there is a tree on the right” “the wind is blowing left to right.”
Club selection	Selecting a club for the shot in hand	“I’m using a driver,” “I’m using a 7 iron.”
Planning	Referring to a plan of action for a shot	“I’m aiming for the left edge of the green,” “hit firm at the hole”
Technical instruction	Specified technical aspects of the motor performance	“Arms bent,” “feet are parallel”
Shot evaluation	Reflecting on and evaluating a shot	“It broke at the end,” “good putt,” “I’m on the green.”
Pre-performance routine*^a^*	Any sequence of task relevant thoughts or actions engaged in systematically prior to a shot.	“Just using my pre-performance routine,” “one, two, three, putt.”
Dwelling*^b^*	Reference to a previous shot played during that round of golf.	“Ahh I wish I had holed that putt on the third,” “if I hadn’t of sliced that driver, I would be level par now.”

*^a^Study 1 only. ^b^Study 2 only.*

Data was split into wood/iron shots and putting, with each type of shot analyzed separately. High and low skilled golfers were compared on the number of thoughts that were verbalized per shot using Mann–Whitney tests, while Wilcoxon tests were used for within person comparisons between number of thoughts verbalized per shot for putts versus wood/iron shots. To analyze the content of verbalizations the percentage of shots where a theme was verbalized was calculated and Mann–Whitney tests compared differences between high and low skilled golfers. Mann–Whitney tests were used due to the small sample size and non-normal distribution of data. Cohen’s (1994) δ effect sizes were calculated to establish the magnitude of differences between high and low skilled golfers.

### Results

#### Number of Thoughts

When playing wood/iron shots significantly more thoughts per shot (*U* = 3.00, *P* = 0.02, δ = 2.07) were verbalized by high skill golfers (*M* = 4.25, *SD* = 0.71) than low skill golfers (*M* = 2.81, *SD* = 0.69). When putting (*M* = 1.83, *SD* = 0.67) there were fewer thoughts per shot compared to wood/iron shots (*M* = 3.52, *SD* = 0.67), this difference was significant (*Z* = 3.05., *P* = 0.002, δ = 2.38). High skill golfers (*M* = 2.19, *SD* = 0.65) had more thoughts per putt than low skill golfers (*M* = 1.47, *SD* = 0.42), although the difference was not significant (*U* = 8.00, *P* = 0.11, δ = 1.34). All effect sizes were large (Cohen, 1994) and ranged from δ = 1.34–2.38.

#### Content of Verbalizations

**FIGURE 1:**
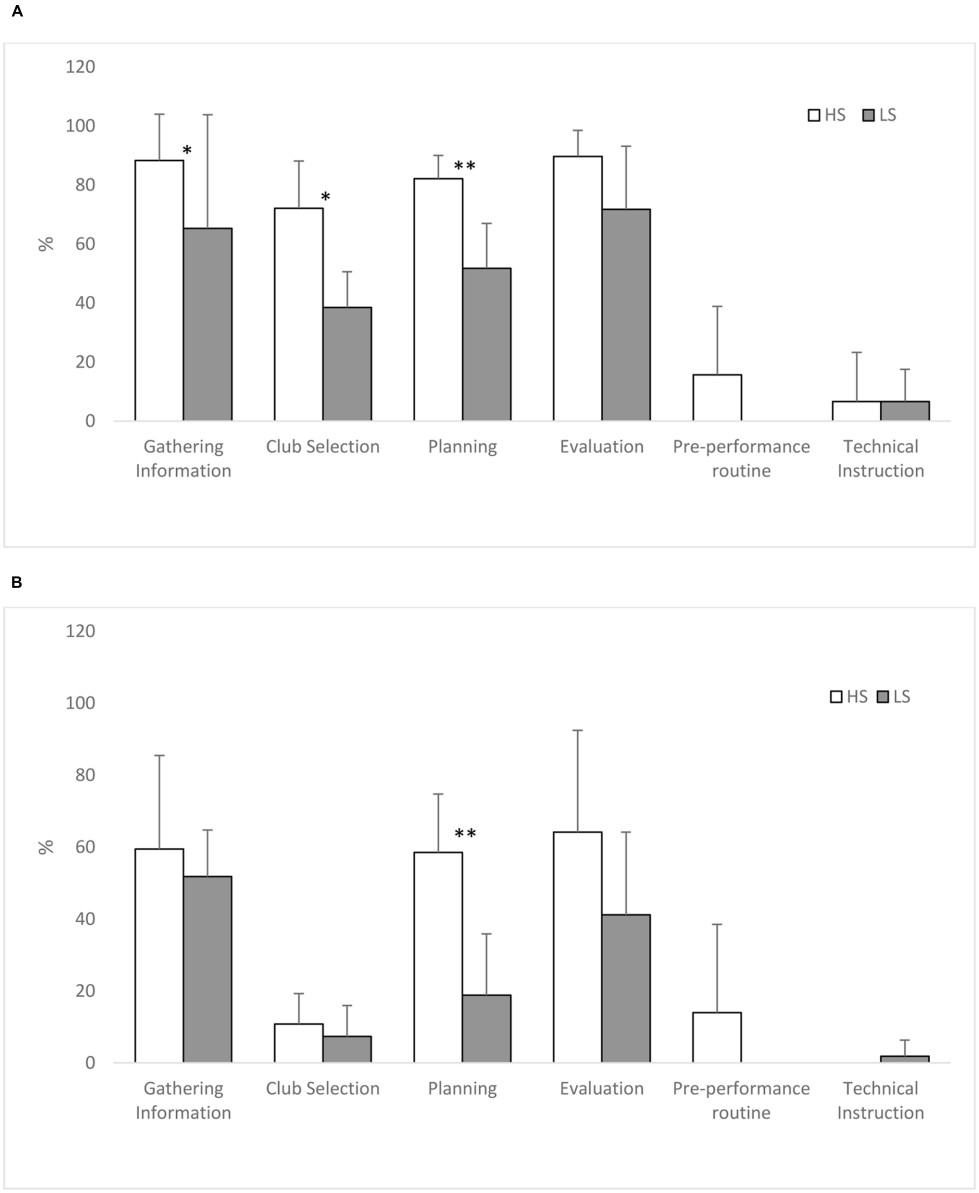
**Percentage of shots where a theme was verbalized (Mean and SD) for High (HS) and Low Skill (LS) golfers when playing Woods/irons (A) and Putting (B). (A)** Significant Skill differences ^∗^*P* < 0.05, ^∗∗^*P* < 0.01. **(B)** Significant Skill differences ^∗^*P* < 0.05, ^∗∗^*P* < 0.01.

For wood/iron shots high skill golfers Gathered information on more shots (88 vs. 65%, *U* = 4.50, *P* = 0.03, δ = 0.84), considered Club selection on more shots (72 vs. 39%, *U* = 3.00, *P* = 0.02, δ = 2.39) and used Planning on more shots (82 vs. 52%, *U* < 0.001, *P* = 0.004, δ = 2.63) compared to the low skill golfers (see **Figure 1A**). When putting high skill golfers verbalized about Planning on a greater proportion of putts than low skilled golfer (59 vs. 19%, *U* = 2.00, *P* = 0.01, δ = 2.38) (see **Figure 1B**). All effect sizes were large (Cohen, 1994) and ranged from δ = 0.84–2.63.

### Discussion

The aim of this study was to investigate the difference in the decision making process between high and lower skilled golfers over six full holes of golf using the TA methodology. Higher skilled golfers provided more verbalizations per shot taken than lower skilled golfers. As predicted higher skilled golfers verbalized more about planning shots for both wood/iron shots and putting, with effect sizes large in both cases. Contrary to predictions there was no evidence of higher skilled golfers providing more verbalizations in the evaluation of a shot, in addition lower level golfers did not verbalize more technical thoughts. The results of the present study were consistent with previous findings by Calmeiro and Tenenbaum (2011) who also found that experienced players verbalized more about planning their shot than lower skilled golfers when putting on an artificial surface. HS participants in the current study also used more planning strategies and goals which guided the execution of a shot. This is also consistent with research by McPherson (2000) who found that experienced, higher level tennis players planned their actions based on sophisticated action plans whereas novices rarely planned. These findings provide support for previous research and theory (Fitts and Posner, 1967; Beilock et al., 2002) that argues that performance in higher skilled performers in the autonomous phase of skill learning is characterized by less explicit cognition toward technique elements of motor performance.

The present study demonstrates clear differences between the thought patterns of high and low skill golfers with large effect sizes evident. It is clear that higher skill golfers focus more on planning their shots and identifying appropriate strategies to reach their desired goal. To our knowledge this is the first study which has applied TA to a more ecological valid environment as a real life golf course. However, due to the current study being conducted in a low pressure environment it is important to develop this area of research even further by looking at expertise differences in different environments such as competitive situations, as sport competitions are situational contexts that induce pronounced stress, even in elite athletes (Holt and Dunn, 2004; Gould and Maynard, 2009).

## Study 2

The aim of Study 2 was to investigate whether stress through the introduction of a competition with monetary prizes influenced the thought process of golfers of differing levels of skill. There is evidence that reinvestment (Masters, 1992) may provide an explanation for underperforming when exposed to pressure, however, few studies have examined thought process during times of pressure situations and how this influences the decision making process. Masters (1992) proposed that during a stressful event a high ability individual in the autonomous stage of learning may experience self-directed attention, which may cause a performer to reinvest thoughts about technique. This inward focus may lead to a breakdown of automaticity since the performer tries to consciously control task performance. For a lower ability performer who is at the cognitive or associative stage of learning this change in cognition under stress would be less pronounced since lower skill performers consciously attend to technical aspects of performance even in low stress situations. Study 2 employs TA to examine changes in the cognition of eight high and eight lower skilled golfers when playing six holes of golf under pressure. In line with the theory of reinvestment (Masters, 1992; Masters and Maxwell, 2008) it was predicted that under stress higher skill golfers would verbalize more thoughts about technique in comparison to practice conditions, while lower skill golfers would not show this same change in cognition.

A second aim was to investigate if measures of propensity for reinvestment relate to greater focus on technique when under stress. Individual differences in propensity for reinvestment can be measured by the movement specific reinvestment scale (Masters et al., 2005) or the decision specific reinvestment scale (Kinrade et al., 2010). Higher scores on the movement specific reinvestment scale have been linked with less efficient motor performance of surgeons on a laparoscopy task when under time pressure (Malhotra et al., 2012), but no research has examined if this scale relates specifically to changes in cognition under stress on a sport task. The decision specific reinvestment scale has been shown to predict performance breakdown under pressure on a computer-based complex basketball decision making task (Kinrade et al., 2015), but research on cognition on real sport tasks is lacking. It was predicted that higher scores on both the movement specific reinvestment scale and decision specific reinvestment scale would relate to more verbalization of technical thoughts when under pressure.

### Method

#### Participants

Participants were eight male high skill golfers (*M* age: 17.50, *SD* = 1.19; M handicap: 2.25, *SD* = 1.75), and eight moderate skill golfers (seven male, one female; *M* age: 17.25, *SD* = 0.46; *M* handicap: 9.62, *SD* = 0.91) who were all members of the same golf club. It is important to note participants in Study 2 were not involved in Study 1. The study and protocol was approved by the University of Central Lancashire ethics committee written consent was provided prior to participation in the study.

#### Materials

Participants played with their own golf clubs on the same six holes of the same golf course in North West England. The six holes were all par 3. As in Study 1 participant’s verbalizations were recorded using a Sennheiser USA ENG G3 wireless digital voice recorder.

Each golfer completed the Decision Specific Reinvestment Scale (DSRS; Kinrade et al., 2010). The 13-item DSRS assesses an individual’s predisposition for exerting conscious control over their decision-making process and consists of two factors. Decision reinvestment assesses a respondent’s tendency to consciously monitor the processes leading up to the decision whereas rumination measures the tendency to reflect upon previous poor decisions. The DSRS is scored on a scale from 0 to 5, with 0 being extremely uncharacteristic to 5 being extremely characteristic. Good reliability has been shown (Cronbach α = 0.89 and 91 for the reinvestment and rumination factors respectively). In addition the scale has shown adequate factorial structure (Kinrade et al., 2010).

#### Procedure

Participants were initially briefed about the study and asked to complete the DSRS (Kinrade et al., 2010). The study involved performing the same six holes of golf on two separate occasions separated by a week. On one occasion a practice round was completed, and on the other occasion a competition round was completed. The order that conditions were performed was counterbalanced. The competition was run as a stroke play event, with scores adjusted for handicap. A presentation ceremony took place at the end of the competition with prizes for the top three performers of £100, £70 and £30. The pressure manipulation phase of this study was similar to previous work by Vine and Wilson (2010) and Vine et al. (2011). They created cognitive anxiety through setting up a competition whereby participants were informed that the individuals with the best performance would receive a £50 prize (in our case £100 for first, £70 for second and £30 for third place). Similarly, in the current study participants were told the competition was built into their curriculum. As they were all part of a further education golf college their lecturer made them aware that this was a competition and it would replace their normal timetable on the specific days. Participants were notified that their scores would be presented back to the whole class the following week to be reviewed. As in Study 1, participants were instructed on how to conduct TA and took part in a series of three TA exercises prior to each round. Each of the golfers played each round of six holes accompanied by the experimenter and were instructed to verbalize their thoughts continuously throughout the six holes apart from when they were executing their shot. If they were silent for a period of longer than 20 s they were asked to resume thinking aloud.

#### Data Analysis

The same method of data transcription and thematic coding used in Study 1 was applied. The coding scheme was modified slightly, with ‘dwelling’ added to the coding scheme due to this emerging frequently in this specific data set. Examples of dwelling from the transcribed verbalizations included “Ahh I wish I had holed that putt on the 3rd,” “If I hadn’t of sliced that driver, I would be level par now.” Pre-performance routine was removed from the coding scheme due to the absence of this theme in this data set. The second author independently analyzed a 10% sample of the raw data. The inter-rater agreement was 95%.

As in Study 1 wood/iron shots and putts were analyzed separately. High and moderate skill golfers were compared on the number of thoughts per shot during practice and then competition using Mann–Whitney tests, and within person comparisons between practice and competition for high skill golfers and moderate skill golfers were made using Wilcoxon tests. The content of verbalizations was analyzed by calculating the percentage of shots where each theme was verbalized, with Mann–Whitney tests used to analyze skill level differences for each theme during practice and then during competition, and Wilcoxon tests used to analyze within person differences for verbalizations made during practice and competition for high level golfers and for moderate skill golfers. Cohen’s (1994) δ effect sizes were calculated to establish the magnitude of each effect. Finally, to analyze if propensity for reinvestment was associated with more thoughts about technical aspects of a shot, Spearman’s correlations were conducted between the decision reinvestment and rumination scores from the DSRS and the percentage of shots where technical thoughts were verbalized. Again, this was done separately for putts and wood/iron shots, and for separate practice and competition conditions for both high and intermediate level performers.

### Results

#### Number of Thoughts

High skilled golfers tended to verbalize more thoughts per shot than moderate skilled (MS) golfers. This was the case for putts in both practice (*U* = 5.50, *P* = 0.005, δ = 1.82; HS: *M* = 2.68, *SD* = 0.47; MS: *M* = 1.85, *SD* = 0.44) and competition (*U* = 13.50, *P* = 0.05, δ = 1.21; HS: *M* = 2.61, *SD* = 0.56; MS: *M* = 1.96, *SD* = 0.51), and for woods/irons in practice (*U* = 9.50, *P* = 0.02, δ = 0.65; HS: *M* = 3.82, *SD* = 0.52; MS: *M* = 3.03, *SD* = 0.72) but not in competition (*U* = 19.50, *P* = 0.16, δ = 0.62; HS: *M* = 3.38, *SD* = 0.47; MS: *M* = 3.02, *SD* = 0.69). There was no difference in number of thoughts verbalized per shot between practice and competition for moderate skill golfers when putting (*Z* = 0.63, *P* = 0.53, δ = 0.02) or playing wood/iron shots (*Z* = 0.28, *P* = 0.78, δ = 0.22). For high skill golfers, there were more thoughts per shot in practice than competition when playing wood/iron shots (*Z* = 1.96, *P* = 0.05, δ = 0.74), but no difference when putting (*Z* = 0.34, *P* = 0.74, δ = 0.14). All significant differences also showed large effect sizes ranging from δ = 0.65 to 1.82 (Cohen, 1994).

#### Content of Verbalizations

**FIGURE 2:**
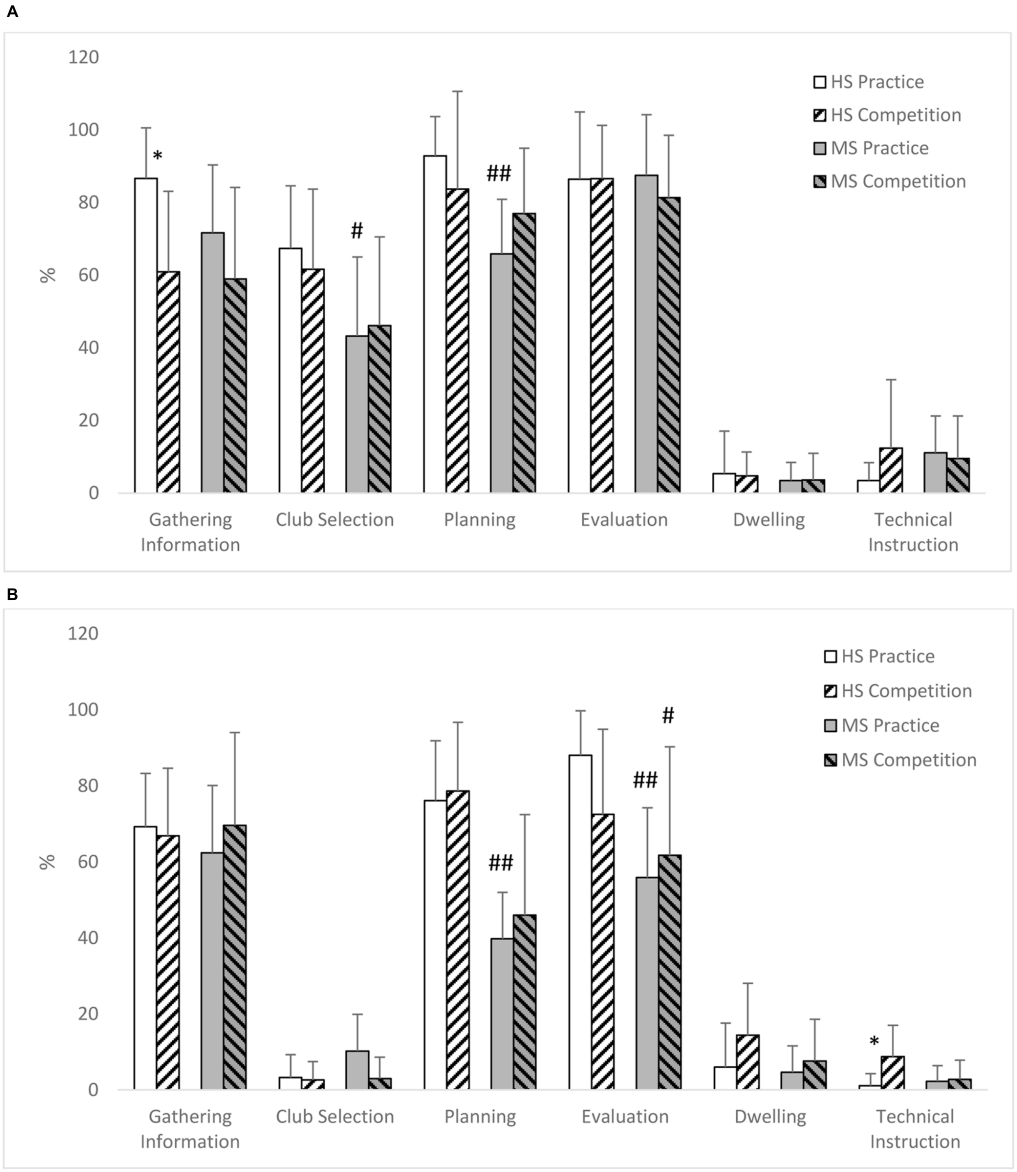
**Percentage of shots where a theme was verbalized (Mean and SD) for High (HS) and Moderate Skill (MS) golfers in Practice and Competition when playing Woods/irons (A) and Putting (B). (A)** Significant Condition differences ^∗^*P* < 0.05. Significant skill differences #*P* < 0.05, ##*P* < 0.01. **(B)** Significant condition differences ^∗^*P* < 0.05. Significant skill differences #*P* < 0.05, ##*P* < 0.01.

**Figure 2** shows the percentage of shots where each theme was verbalized during putting and wood/iron shots for high and moderate skill golfers in both practice and competition. To analyze skill level differences, the verbalizations of high and moderate skill golfers were compared for the practice round and then for the competition round using Mann–Whitney tests. During the practice round significant differences were found for the themes Club selection (*U* = 12, *P* = 0.03, δ = 1.23) and Planning (*U* = 5.00, *P* = 0.004, δ = 2.08) for wood/iron shots and Planning (*U* = 2.00, *P* = 0.002, δ = 2.61) and Evaluation (*U* = 2.00, *P* = 0.002, δ = 2.13) for putting. High skill golfers verbalized more thoughts than moderate skill golfers about Club selection (67 vs. 43%) and Planning (93 vs. 66%) on wood/iron shots and more thoughts about Planning (76 vs. 40%) and Evaluation (88 vs. 56%) when putting. For the competition round, the only significant difference found was for Planning during putting (*U* = 10.0; *P* = 0.02, δ = 1.46), with high skill golfers using Planning on more putts than the moderate skill golfers (79 vs. 46%). All significant differences showed large effect sizes ranging from δ = 1.23 to 2.61 (Cohen, 1994).

To analyze if playing in competition influenced thoughts, differences in verbalizations were compared between the practice and competition conditions for the high skill golfers and then moderate skill golfers separately using Wilcoxen tests. For high level golfers there was a significant difference in the theme Gathering information (*Z* = -2.03, *P* = 0.02, δ = 1.16) when hitting wood/iron shots, with gathering information verbalized on more shots in practice than competition (87 vs. 61%). A significant difference was also found in the theme Technical instruction during putting (Z = -2.03, *P* = 0.04, δ = 1.14), with more technical instruction used during competition than practice (9 vs. 1%). Effect sizes were large in both comparisons (Cohen, 1994). For moderate skill golfers no significant differences were found for any theme between practice and competition.

#### Correlation Analysis

To establish if propensity for reinvestment was associated with golfers verbalizing more thoughts about technical aspects of a golf shot during competition a correlation analysis was conducted. The difference score for percentage of shots where technical instruction was verbalized in competition minus practice was calculated for each participant for both putts and wood/iron shots. A higher difference score would indicate more verbalizations of technical instructions in competition compared to practice, hence suggesting reinvestment when playing in a competition. Spearman correlations were conducted between technical instruction difference scores, the two factors of the DSRS (decision reinvestment and decision rumination), and the two factors of the MSRS (conscious motor processing, self-consciousness). This was done separately for wood/iron shots and putts for both high and moderate skill golfers (see **Table 2**).

**Table 2:** Descriptive statistics and Spearman correlation coefficients for measures of reinvestment and technical instruction.

Variable	Mean (*SD*)	1	2	3	4	5
**High skill**						
(1) TI putting^cpd^	7.63 (8.55)					
(2) TI wood/irons^cpd^	8.88 (20.74)	0.71^∗^				
(3) Decision reinvestment	12.50 (3.25)	0.85^∗∗^	0.29			
(4) Decision rumination	13.63 (2.07)	-0.04	0.14	-0.05		
(5) Self-consciousness	35.25 (6.84)	0.04	0.24	-0.08	-0.33	
(6) Conscious motor processing	15.75 (4.03)	0.31	0.44	0.19	-0.04	0.90^∗∗^
**Moderate skill**						
(1) TI putting^cpd^	0.50 (5.18)					
(2) TI wood/irons^cpd^	-1.63 (18.88)	0.03				
(3) Decision reinvestment	10.63 (2.87)	0.07	0.70			
(4) Decision rumination	16.50 (4.11)	-0.02	0.08	0.29		
(5) Self-consciousness	35.88 (5.11)	-0.07	-0.17	0.24	-0.47	
(6) Conscious motor processing	15.80 (4.83)	-0.27	-0.29	0.20	-0.43	0.93^∗∗^

*TI putting^cpd^ is the difference score for percentage of putting shots where technical instruction is verbalized in competition minus practice. TI wood/irons^cpd^ is the difference score for percentage of wood/iron shots where technical instruction is verbalized in competition minus practice. ^∗^P < 0.05, ^∗∗^P < 0.01.*

##### Decision specific reinvestment

For high skill golfers it was found that decision reinvestment had a strong positive relationship with technical instruction verbalizations when putting (*r*_s_ = 0.84, *p* = 0.008), but only a weak relationship for wood/iron shots (*r*_s_ = 0.29, *p* = 0.49). For moderate skill participants decision reinvestment had a strong positive relationship with technical instruction verbalizations during wood/iron shots (*r*_s_ = 0.70, *p* = 0.05), but not for putts (*r*_s_ = 0.07, *p* = 0.87). Decision rumination was not related to technical instruction for either skill level on any type of shot.

##### Movement specific reinvestment

For high skill performers conscious motor processing was moderately positively correlated with technical instruction verbalizations for wood/irons (*r*_s_ = 0.44, *p* = 0.27) and for putts (*r*_s_ = 0.31, *p* = 0.46), although in each case the relationship was non-significant. There were no clear relationships between conscious motor processing and technical instructions verbalized on either putts or woods/irons for moderate skill golfers. Self-consciousness was not related to technical instructions for either the high skill or moderate skill group on any shot type. There was a strong positive relationship between conscious motor processing and self-consciousness for both high (*r*_s_ = 0.90, *p* = 0.002) and moderate skill golfers (*r*_s_ = 0.93, *p* = 0.001).

### Discussion

Study 2 aimed to investigate whether the introduction of competitive pressure influenced performance and thought process in high and moderate skill golfers. Results support the main hypothesis, under competitive pressure HS golfers were more likely to verbalize technical rules and refer to step-by-step mechanics of their swing in comparison to normal practice conditions as indicated by a large effect size, while this change in cognition was not apparent for lower skill golfers. This finding was most pronounced when putting than when playing wood iron shots. Such findings are line with the theory of reinvestment (Masters, 1992; Masters and Maxwell, 2008), since a breakdown in cognition under pressure is only predicted for higher skill performers who are at the autonomous stage of learning (Fitts and Posner, 1967) and would normally perform a motor task automatically without conscious thought of movement processes. When under pressure there is an inward shift in attention toward the control of the body in an attempt to consciously control task performance.

The second aim of the study was to investigate if measures of propensity for reinvestment related to greater focus on technique when under stress. Findings showed that higher scores on decision reinvestment subscale of the DSRS were strongly associated with more technical thoughts during performance for high skill performers when putting and for low skill performers when playing wood and iron shots. Measures of movement specific reinvestment were not associated with changes in thought patterns under competitive pressure. This finding extends the work of Kinrade et al. (2015) who showed that performance breakdown on a simulated basketball decision making task was better predicted by the DSRS than the original reinvestment scale of Masters et al. (1993).

This study presented a number of important findings. First, when faced with the pressure of a competition higher skill golfers’ thought process changed and regressed to a less automatic and more technical step-by-step process. Secondly, clear differences in the thought processes of high and intermediate level golfers during both practice and competition were found, with less planning of shots conducted by lower skill golfers. Finally, propensity for decision reinvestment was a strong correlate of changes in cognition toward a more technique focus when under competitive pressure.

## General Discussion

The current study has progressed previous research around cognitive processing and expertise within sport by moving away from laboratory based artificial studies and moving into more ecologically valid environments where cognition is measured during the performance of a real sport task using TA. Findings provide evidence of skill level differences in cognition during sport performance, and changes in cognition as a result of competitive pressure among high skill performers.

We present evidence to support Fitts and Posner’s (1967) stage model of motor learning. At later stages of learning performance was guided by procedural knowledge where appropriate strategies were used to achieve the desired goal. We also present evidence to support Masters (1992) theory of reinvestment. When high skill golfers were put in a pressurized competitive environment we found evidence of reinvestment, with more verbalizations about technical elements of a shot than in a low pressure environment. This suggests that under stress higher skill performers at a later stage of learning may regress in their thoughts to an earlier stage of learning where conscious control of motor performance is more prevalent. This finding was strongest for performers scoring high on the decision reinvestment subscale of the DSRS (Kinrade et al., 2010), and suggests this may be a useful tool for identifying people at greater risk for reinvestment in high pressure environments.

This paper has provided a significant original contribution to the current sport psychology literature by providing an understanding of differing skill level golfers thought process within real time and in an ecologically valid environment. The paper further advances our understanding of cognitive process during performance as much of the current and previous literature investigating cognitive differences has been conducted in a laboratory setting or used retrospective methods. In addition, we have established the DSRS may be used to identify people more prone to internalize thoughts and reinvest during competition, and thus may be of use to coaches and sport psychologists.

Although this paper has provided a significant contribution to the current sport psychology literature it is important to acknowledge its limitations. One limitation is the assumption that the higher level golfers are in the autonomous phase of learning as this was not tested before data collection commenced (Fitts and Posner, 1967). Future studies might consider having a larger range of abilities (golf handicaps). For example, using participants with lower handicaps in comparison to those who have never played golf before (true novices). It is important to acknowledge the relatively small sample sizes and limited statistical power of the analyses, however, large effect sizes were consistently found for the differences in verbalizations of higher and lower skilled golfers and differences between practice and competition conditions. The sample size is still a progression and improvement in comparison to similar previous research. For example, Calmeiro and Tenenbaum’s (2011) ‘experienced’ group consisted of three participants with a mean handicap of 10.3, whereas the current study had six participants in the experienced group with a mean handicap of four. Furthermore, Study 2 aimed to put differing levels of golfers under higher levels of pressure through their participation in a competitive situation. It is recommended that future research examines the extent to which these performers perceive the competition as a stressful or anxious situation and one way of doing that would be for participants to complete a pre-performance questionnaire such as the Competitive State Anxiety Inventory- 2R (Cox et al., 2003). In addition attaching heart rate monitors to participants and collecting salivary cortisol samples (Coetzee, 2011) during practice and competition would also provide a physiological variable which could be used to measure stress levels.

A further limitation of the work is that decision making is not always a conscious process and TA cannot assess what happens to the decision making process outside of awareness (Bowers et al., 1990; Jacoby et al., 1992; Wegner, 1994). That being said, both studies were aiming to identify what the performer consciously attends to and uncover the differences between two skill levels.

Study 2 used a counterbalancing design. Such a design might result in an alteration in the meaning of practice for those who engaged in the competition round first. Although we believe this is the most appropriate design for this type of studies future studies could examine qualitatively the perceptions of the golfers on this issue.

An important issue for future research would also to consider the influence of gender. There is evidence that males and females cope differently with stressful encounters in sport (Nicholls and Polman, 2007). However, it is unclear whether differences exist in decision making and how stress might influence this process for male and female athletes.

## Conclusion

Collective findings have provided further support for the cognitive differences between differing levels of golfers. Through using TA our studies have been able to collect rich verbal data from differing levels of golfers and provide clear differences between high, moderate and low skilled performers. Furthermore, through the use of TA we have also been able to identify how a stressful environment such as a competition can change a performer’s thought processes depending on their level of expertise. We have also shown that changes in decision making under pressure are strongly associated with propensity for reinvestment as measured by the DSRS (Kinrade et al., 2010).

## Author Contributions

All authors of this paper made an equal contribution.

## Conflict of Interest Statement

The authors declare that the research was conducted in the absence of any commercial or financial relationships that could be construed as a potential conflict of interest.
